# Fetal biometry reference ranges derived from prospective twin population and evaluation of adverse perinatal outcome

**DOI:** 10.1002/uog.29190

**Published:** 2025-02-27

**Authors:** P. Dicker, S. Daly, R. M. Conroy, F. M. McAuliffe, M. P. Geary, J. J. Morrison, S. S. Carroll, F. D. Malone, F. M. Breathnach

**Affiliations:** ^1^ Department of Obstetrics and Gynaecology Royal College of Surgeons in Ireland Dublin Ireland; ^2^ Rotunda Hospital Dublin Ireland; ^3^ Department of Epidemiology & Public Health Royal College of Surgeons in Ireland Dublin Ireland; ^4^ UCD Perinatal Research Centre University College Dublin Dublin Ireland; ^5^ National Maternity Hospital Dublin Ireland; ^6^ Department of Obstetrics and Gynaecology University of Galway Galway Ireland; ^7^ University Hospital Galway Galway Ireland

**Keywords:** fetal growth, reference ranges, twin pregnancy

## Abstract

**Objectives:**

Ultrasound‐derived estimates of fetal size play an integral role in the prenatal management of twin pregnancy. These biometric measurements are conventionally plotted against singleton standards. We sought to establish fetal growth references for abdominal circumference, head circumference, biparietal diameter, femur diaphysis length and estimated fetal weight (EFW) in twin pregnancy. We also aimed to determine whether the performance of a twin fetal growth reference was superior to a singleton reference in the prediction of adverse perinatal outcome in twin pregnancies.

**Methods:**

This was a retrospective analysis of data collected prospectively in the Evaluation of Sonographic Predictors of Restricted growth in Twins (ESPRiT) study, which was conducted at eight academic perinatal centers in Ireland, all with tertiary neonatal intensive care facilities. Only diamniotic twin pregnancies with two live fetuses were eligible for inclusion. Exclusion criteria were monoamnionicity, congenital abnormality, twin‐to‐twin transfusion syndrome or previable fetal demise (< 24 weeks' gestation). Using serial ultrasound observations, we applied fractional polynomial multilevel models to derive an equation for fetal centile determination. We compared these centiles with published singleton and twin fetal references, with particular focus on the Fetal Medicine Foundation (FMF) references. Using the last ultrasound examinations before delivery, we determined associations between biometric measures and a composite measure of adverse perinatal outcome (intraventricular hemorrhage, periventricular leukomalacia, hypoxic ischemic encephalopathy, necrotizing enterocolitis, bronchopulmonary dysplasia, sepsis or perinatal death), neonatal intensive care unit admission, preterm delivery (< 34 weeks) and birth‐weight discordance ≥ 25%, based on the varied prevalence of these outcomes. We compared our results with the singleton and twin FMF reference ranges and the twin reference of the Southwest Thames Obstetric Research Collaborative (STORK) study.

**Results:**

Among the 948 twin pairs that met the inclusion criteria, 776 (81.9%) dichorionic and 172 (18.1%) monochorionic twin pairs completed the prospective 2‐weekly ultrasound surveillance program. Fetal biometric measurements were obtained in 15 274 ultrasound assessments (12 279 in dichorionic and 2995 in monochorionic twin pairs) from serial ultrasound assessments. The median number of ultrasound assessments per pregnancy was 8 (interquartile range, 7–9). Growth trajectories in this cohort were consistent with the FMF and STORK published twin cohorts and notably less consistent with the FMF singleton standard. Compared with the FMF singleton standards, the 50^th^ centiles for twins were greater early in pregnancy and lower later in pregnancy for all biometric measures, in both dichorionic and monochorionic twin pregnancies. This crossover in growth occurred at approximately 28 weeks' gestation for dichorionic twins and earlier for monochorionic twins. The 50^th^ centiles for EFW were comparable to the FMF twin standards for both monochorionic and dichorionic twins, but with lower 10^th^ centiles for dichorionic twins in the third trimester. The current (ESPRiT) twin reference ranges, the STORK twin reference ranges and the FMF twin reference ranges showed larger and statistically significant (*P* < 0.01) odds ratios for multiple biometric measures and multiple adverse perinatal outcomes, for both monochorionic and dichorionic twins, not observed with the FMF singleton reference standard.

**Conclusions:**

In this analysis of data from the prospective ESPRiT cohort study, we confirm significant differences between twin fetal growth patterns and singleton standards, consistent with previous studies. Our results also offer some validation of the new FMF reference for EFW in twins. The outcome‐based evidence from this study suggests that a twin‐specific growth reference should be used in preference to a singleton chart for fetal growth evaluation in twin pregnancy. © 2025 The Author(s). *Ultrasound in Obstetrics & Gynecology* published by John Wiley & Sons Ltd on behalf of International Society of Ultrasound in Obstetrics and Gynecology.

## INTRODUCTION

Assessment of fetal size is one of the cornerstones of prenatal care. Owing to many advances in ultrasound technology in recent years, optimal images are more readily attainable for basic ultrasound biometry, in particular because of improvements in resolution and image magnification. Accurate evaluation of fetal biometry has become increasingly important as a key determinant that drives the need for iatrogenic preterm delivery. Gestational‐age‐related reference intervals for estimated fetal weight (EFW) are used commonly for predicting likely fetal health and pregnancy management, by comparing individual and composite fetal biometric measurements with a reference population^1^.

Published reference charts^2^, ^3^, ^4^, ^5^, ^6^, ^7^, ^8^, ^9^, ^10^, ^11^, ^12^, ^13^ for singleton pregnancy have been derived from a variety of geographic or ethnic populations. However, different study designs (cross‐sectional *vs* cohort), varied inclusion criteria and disparate modeling strategies may all contribute to inaccurate centile calculation. Specific references have been proposed by the Fetal Medicine Foundation (FMF)^9^, ^12^ and the World Health Organization (WHO)^13^.

Most studies have been conducted to derive reference charts for fetal size in singleton gestations. In contrast, few studies on fetal biometric measurements have been conducted on multiple gestations^14^, ^15^, ^16^, ^17^, ^18^, ^19^, ^20^, ^21^, ^22^, ^23^, ^24^, ^25^ and the lack of supporting outcome‐based evidence has led to the widespread practice of using singleton charts to assess fetal growth in twins. It is possible that the application of published reference charts determined in singletons may result in under‐ or overestimation of twin size, with consequences for perinatal decision‐making.

The objective of this study was to construct reference charts for fetal growth biometry using longitudinal assessments from an unselected prospectively recruited twin cohort and to compare these serial data with other published references, both singleton and twin. We further sought to evaluate the utility of twin‐specific references compared with a singleton reference in the prediction of adverse perinatal outcome in twin pregnancy.

## METHODS

### Study design

This was a retrospective analysis of data collected prospectively from a consecutive cohort of 1028 unselected twin pregnancies enrolled in the Evaluation of Sonographic Predictors of Restricted growth in Twins (ESPRiT) study, a multicenter study conducted at eight academic perinatal centers in Ireland, all with tertiary neonatal intensive care facilities, from May 2007 to October 2009. Institutional review board approval was obtained at each participating site, and the study participants gave written informed consent. Dedicated site‐specific sonographers were given study‐specific training. The ESPRiT study eligibility criteria were twin pregnancy from 11 + 0 to 22 + 0 weeks' gestation with both fetuses alive at the time of enrollment and with intact membranes. The study exclusion criteria were monoamnionicity, a major structural abnormality in either twin, twin‐to‐twin transfusion syndrome, fetal aneuploidy (either suspected or confirmed) or previable fetal demise (< 24 weeks' gestation). After obtaining informed consent on the study objectives and procedures, participant demographic data and ultrasound data were recorded on the ViewPoint ultrasound archiving system (MDI ViewPoint, Jacksonville, FL, USA).

Chorionicity was assigned according to the presence or absence of an extension of placental tissue into the base of the intertwin membrane, visualized on ultrasound scans as the lambda sign or T‐sign, respectively. Chorionicity was recorded at the enrollment scan, before 14 weeks' gestation, and confirmed on placental pathology.

For participants who were uncertain of their menstrual dates or had a non‐classical menstrual history, gestational age was determined by the biometry of the larger fetus. Gestational age was also derived from the biometry of the larger fetus if there was a discrepancy of more than 5 days between crown–rump length and menstrual data‐derived gestational age, before 14 weeks' gestation.

A complete fetal anatomical survey was conducted between 18 and 20 weeks' gestation for all participating pregnancies. Where either twin was found to have a major structural malformation or confirmed aneuploidy, that case was excluded from this particular protocol for gathering biometric data. Two‐weekly assessment of fetal biometry was scheduled from 24 weeks' gestation onwards for dichorionic twins and from 16 weeks' gestation for monochorionic pairs. Ultrasound assessments (GE Voluson 730 Expert; GE Healthcare, Zipf, Austria) of outer‐to‐inner biparietal diameter (BPD), head circumference (HC), abdominal circumference (AC) and femur diaphysis length (FL) were obtained at each visit. The EFW was calculated at each assessment using the Hadlock formula^26^. The four‐measure Hadlock formula was used (BPD, HC, AC, FL) as standard, with use of the three‐measure formula reserved for circumstances in which a particular measure could not be ascertained or was unreliably measured. In the analysis presented here, the Hadlock three‐measure formula (HC, AC, FL) was used to determine a reference range and was used for the comparisons.

At enrollment, each fetus was designated as either Twin 1 or Twin 2, based on proximity of each amniotic sac to the cervix. At each assessment, twin labels were further qualified by recording fetal presentation and position, placental location and fetal gender.

Data were contemporaneously transferred to an ultrasound reporting system (ViewPoint) and uploaded to a web‐based centralized database each week by the study sonographers. The database was assessed regularly throughout the study period using preprogrammed structured queries with continuous feedback loops to the contributing centers for data completeness and verification.

### Fetal growth references

Twin fetal biometry data from the ESPRiT study were used to construct reference charts for AC, HC, FL, BPD and EFW and were compared against the singleton references recommended by the FMF (Snijders and Nicolaides^9^ for HC, AC and FL and Nicolaides *et al*.^12^ for EFW). BPD is not included as part of the FMF growth reference, but, as it was ascertained in our twin population, we included BPD for reference range determination. It was excluded from further analyses that sought comparison with the FMF references. For comparison with twin reference ranges, the twin study by Wright *et al*.^21^, the FMF growth reference for EFW in twins, was of particular interest, owing to its very large study population. The twin fetal‐weight reference developed by Stirrup *et al*.^25^, on behalf of the Southwest Thames Obstetric Research Collaborative (STORK) cohort, was used as a second comparison. The objective of these comparisons was to validate patterns observed previously in other twin studies^21^. For brevity of presentation, comparison with other singleton references was not made.

### Perinatal outcomes

Maternal and fetal outcomes were obtained for all pregnancies. Composite adverse perinatal outcome was defined as the occurrence of any of the following: intraventricular hemorrhage (IVH), periventricular leukomalacia (PVL), hypoxic ischemic encephalopathy (HIE), necrotizing enterocolitis (NEC), bronchopulmonary dysplasia, sepsis or perinatal death. As members of the Vermont Oxford Network, all ESPRiT study sites applied standardized definitions^27^ for IVH, PVL, HIE, NEC, bronchopulmonary dysplasia and sepsis.

Birth‐weight centiles were determined retrospectively using the FMF reference for singletons^12^ and a twin reference based on a UK population^28^.

The optimal cut‐off for birth‐weight discordance, as a predictor of adverse perinatal outcome, was found to be 18% previously^29^, though a cut‐off of 25% is recommended by the International Society of Ultrasound in Obstetrics and Gynecology (ISUOG)^30^, supported by a Delphi consensus that the 25% cut‐off is relevant in defining selective fetal growth restriction^31^. Given that admissions to the neonatal intensive care unit (NICU) were very high in this study, we restricted our attention to those in NICU for at least 10 days, corresponding to the median duration of NICU stay.

The adverse perinatal outcomes considered in this secondary analysis were: (1) composite adverse perinatal outcome; (2) NICU admission (length of stay ≥ 10 days); (3) preterm delivery (< 34 weeks' gestation); and (4) intertwin birth‐weight discordance ≥ 25%. The last ultrasound examination before delivery was used in this analysis as it is considered to be the most reliable and simple method for evaluating adverse perinatal outcomes^31^. Composite adverse perinatal outcome and NICU admission were analyzed for each twin individually (singular twins). The smaller twin, as judged by ultrasound biometry, was used to compare the twin‐pair outcomes of preterm delivery and birth‐weight discordance.

Due to the variable prevalence of different adverse outcomes in twin pregnancy^32^, it is reasonable to consider different centile cut‐offs in twins, compared with those normally considered relevant in a singleton pregnancy^1^. In practice, centile cut‐offs are often lowered to potentially increase sensitivity for rare outcomes, but seldom are they increased above the 10^th^ centile. To address this issue, for the comparison of different biometry reference ranges, we used centile cut‐offs for each biometric measure equal to the prevalence of adverse perinatal outcome occurring in our study sample of monochorionic and dichorionic twins.

### Statistical analysis

Numerical data are summarized with median (interquartile range (IQR)) and categorical data with *n* (%). We report associated odds ratios (OR) and 95% CI for adverse perinatal outcomes and the test positivity rate. The latter represents the number of singular twins that fell under the centile cut‐off, determined by the relevant outcome prevalence, at the last ultrasound examination before delivery. Degrees of statistical significance are highlighted for *P*‐values < 0.01, < 0.001 and < 0.0001. Comparisons in this analysis were restricted to the two largest twin studies published to date, the studies by Stirrup *et al*.^24^, ^25^ on behalf of the STORK cohort (9866 ultrasound examinations) and the study by Wright *et al*.^21^, the FMF twin reference (13 143 ultrasound examinations). The FMF singleton growth references^9^, ^12^ were used as the singleton comparison.

Longitudinal fetal biometry data in twins have a hierarchical structure with three levels of variation: variation between twin pairs (pregnancies), variation between fetuses within a twin pair and residual variation (measurement error). We used fractional polynomial linear mixed models to analyze the fetal biometry data for monochorionic and dichorionic twins separately. Consideration was given to the individualized growth assessment of a subgroup of this population in modeling fetal growth^33^, ^34^. Rossavik growth curves are used in, and more commonly associated with, individualized growth assessment, which seeks to determine growth pathology scores in the third trimester of pregnancy. Their implementation here is different in that they are used to model fetus‐level variation to determine a reference range. Further details of the modeling approach and resulting equations are provided in Appendix S1. Fetal growth curves and reference charts were established for AC, FL, HC, BPD, and EFW. Centiles were calculated using formulas for exact gestational week (+ 0 days).

Using fetal data from the final predelivery ultrasound examination, generalized estimating equations with a robust sandwich estimator were used to determine associations of outcomes with each biometric measure. For twin‐pair‐related outcomes (preterm delivery and birth‐weight discordance), the smaller twin for the biometric measure was used in a logistic regression analysis.

## RESULTS

### Population characteristics

Among 1028 twin pregnancies enrolled in the ESPRiT study, there were 15 pregnancies with congenital anomalies, 24 pregnancies with a previable single or double death, 16 cases of twin‐to‐twin transfusion syndrome, eight pregnancies that delivered outside of a participating study center and 17 participants who withdrew from the study. The remaining 948 twin pregnancies were included in this analysis, of which 172 (18.1%) were monochorionic diamniotic and 776 (81.9%) were dichorionic diamniotic (Table 1). Serial fetal biometric data and pregnancy outcome data were ascertained for all (948/948) included study participants.

**Table 1:** Baseline characteristics of 948 diamniotic twin pregnancies included in study

Characteristic	All twin pregnancies (*n* = 948)	Monochorionic twin pregnancies (*n* = 172)	Dichorionic twin pregnancies (*n* = 776)
Maternal age at presentation (years)	33 (29–37)	32 (28–36)	34 (30–37)
Maternal BMI at presentation (kg/m^2^)	24 (22–28)	24 (22–28)	24 (22–28)
Current smoker	110/905 (12.2)	20/167 (12.0)	90/738 (12.2)
Medical history			
Allergy	26 (2.7)	4 (2.3)	22 (2.8)
Autoimmune disease	3 (0.3)	1 (0.6)	2 (0.3)
Diabetes mellitus	7 (0.7)	2 (1.2)	5 (0.6)
Epilepsy	10 (1.1)	1 (0.6)	9 (1.2)
Gastrointestinal disease	28 (3.0)	1 (0.6)	27 (3.5)
Hypertension	12 (1.3)	0 (0)	12 (1.5)
Infection	14 (1.5)	1 (0.6)	13 (1.7)
Liver disease	1 (0.1)	1 (0.6)	0 (0)
Nephropathy	5 (0.5)	0 (0)	5 (0.6)
Respiratory disease	43 (4.5)	6 (3.5)	37 (4.8)
Parity			
Nulliparous	451 (47.6)	85 (49.4)	366 (47.2)
Parous	497 (52.4)	87 (50.6)	410 (52.8)
Method of conception			
Spontaneous	598/828 (72.2)	140/146 (95.9)	458/682 (67.2)
Assisted	230/828 (27.8)	6/146 (4.1)	224/682 (32.8)
Gestational hypertension/pre‐eclampsia	91 (9.6)	18 (10.5)	73 (9.4)

Data are given as median (interquartile range), *n*/*N* (%) or *n* (%). BMI, body mass index.

A total of 7736 twin pregnancy ultrasound assessments (visits) were obtained for fetal biometry, corresponding to 15 274 individual fetal assessments for each biometric measure (12 279 and 2995 for dichorionic and monochorionic twins, respectively). The median number of visits per study participant was 8 (IQR, 7–9). In total, 55.4% of all study visits were precisely 14 days apart and 88.5% were within a window of 7–21 days, indicating strong adherence to the study protocol. Unavailability of individual fetal biometric measurements was 1.5% for AC, 4.1% for HC, 2.5% for BPD and 2.0% for FL, across the entirety of the database.

The median maternal age at presentation was 33 years (Table 1). The proportion of nulliparous (47.6%) and parous (52.4%) pregnancies was similar across the entire study population. Assisted conception accounted for 4.1% of the monochorionic pregnancies and 32.8% of dichorionic pregnancies. Gestational hypertension or pre‐eclampsia occurred in 9.6% of the study participants.

### Perinatal outcomes

The median gestational age at delivery was 37 weeks, with 14.6% of pregnancies delivering before 34 completed weeks of gestation (Table 2). There were 11 cases of perinatal death, with seven cases (six pregnancies) in monochorionic twins and four cases (three pregnancies) in dichorionic twins, representing a perinatal mortality rate of 35 per 1000 in monochorionic twin pregnancies and 3.9 per 1000 in dichorionic twin pregnancies.

**Table 2:** Perinatal outcomes of 948 diamniotic twin pregnancies included in study

Outcome	All twin pregnancies	Monochorionic twin pregnancies	Dichorionic twin pregnancies
Twin pairs	*n* = 948	*n* = 172	*n* = 776
Gestational age at delivery (weeks)	37 (35–38)	36 (34–37)	37 (36–38)
Preterm delivery (< 34 weeks)	138 (14.6)	42 (24.4)	96 (12.4)
Birth‐weight discordance			
≥ 18%*	205 (21.6)	40 (23.3)	165 (21.3)
≥ 25%	83 (8.8)	17 (9.9)	66 (8.5)
Individual twins	*n* = 1896	*n* = 344	*n* = 1552
Birth‐weight centile			
Singleton reference†	16 (4–40)	13 (4–34)	17 (4–41)
Twin reference‡	62 (36–82)	62 (39–82)	62 (35–82)
NICU admission	809 (42.7)	185 (53.8)	624 (40.2)
NICU admission ≥ 10 days	394 (20.8)	106 (30.8)	288 (18.6)
Perinatal death	11 (0.6)	7 (2.0)	4 (0.3)
HIE	2 (0.1)	1 (0.3)	1 (0.1)
IVH	26 (1.4)	10 (2.9)	16 (1.0)
PVL	3 (0.2)	0 (0)	3 (0.2)
NEC	10 (0.5)	3 (0.9)	7 (0.5)
Bronchopulmonary dysplasia	250 (13.2)	63 (18.3)	187 (12.0)
Bronchopulmonary dysplasia requiring ventilation	178 (9.4)	48 (14.0)	130 (8.4)
Sepsis	100 (5.3)	32 (9.3)	68 (4.4)
Composite adverse perinatal outcome§	286 (15.1)	80 (23.3)	206 (13.3)

Data are given as median (interquartile range) or *n* (%).

* Breathnach *et al*.^29^.

† Nicolaides *et al*.^12^.

‡ Briffa *et al*.^28^.

§ Intraventricular hemorrhage (IVH), periventricular leukomalacia (PVL), hypoxic ischemic encephalopathy (HIE), necrotizing enterocolitis (NEC), bronchopulmonary dysplasia, sepsis or perinatal death. NICU, neonatal intensive care unit.

The preterm delivery rate for monochorionic twins (24.4%) was twice that of dichorionic twins (12.4%) (Table 2). The median birth‐weight centile using the FMF singleton birth‐weight reference^12^ was 13 (IQR, 4–34) for monochorionic twins and 17 (IQR, 4–41) for dichorionic twins, with 10^th^ centiles of 0.4 and 0.5, respectively. When using a twin birth‐weight reference^28^, the birth‐weight centile was 62 (IQR, 39–82) for monochorionic twins and 62 (IQR, 35–82) for dichorionic twins, with 10^th^ centiles of 17 and 15, respectively.

The rate of birth‐weight discordance ≥ 25% was similar for both chorionicities (9.9% and 8.5%). The overall NICU admission rate (≥ 10 days) was 20.8%, with a higher rate in monochorionic twins (30.8%) than in dichorionic twins (18.6%). The primary reason for NICU admission was prematurity/low birth weight (77.8%). Composite adverse perinatal outcome (IVH, PVL, HIE, NEC, bronchopulmonary dysplasia, sepsis or perinatal death) occurred in 15.1% of all twins, with 23.3% occurring in monochorionic and 13.3% in dichorionic twins. Bronchopulmonary dysplasia was the most common constituent outcome (13.2%).

### Twin fetal biometry centiles

Scatter‐density plots of the fetal biometry assessments for dichorionic and monochorionic twins are presented in Figures S1 and S2, respectively. These illustrate the characteristic growth trajectories apparent in twin pregnancy and reflect the intensity of assessment across gestational age in this study. The ratio of dichorionic twins to monochorionic twins in this study was 4.5 to 1, and this is reflected in the greater number of assessments that can be visualized in Figure S1 relative to Figure S2.

The resulting equations from the statistical modeling were used to determine centiles for each biometric measure. Table S1 (dichorionic twins) and Table S2 (monochorionic twins) present the estimated 10^th^, 50^th^ and 90^th^ centiles for serial fetal biometric measurements in this twin study population. As with other published formulas, they may be used to determine centiles at any gestational age for each biometric measure.

### Comparison with singleton reference ranges

**Figure 1:**
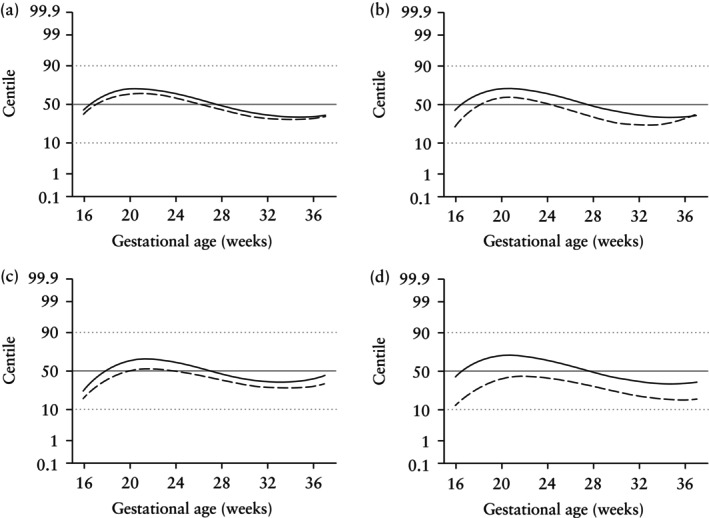
Graphs showing comparison of 50^th^ centiles for abdominal circumference (a), head circumference (b), femur length (c) and estimated fetal weight (d), based on reference range determined in this study (using ESPRiT data) in dichorionic (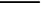) and monochorionic (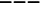) twins, with the FMF singleton reference^9^, ^12^. FMF centiles are shown in gray.

Figure 1 illustrates the 50^th^ centiles for each biometric measure from this population plotted on the FMF singleton references^9^, ^12^. These figures illustrate that, when compared with singleton standards, the observed 50^th^ centiles in twins are higher than the singleton 50^th^ centile early in pregnancy, but lower later in pregnancy for all biometric measures, in both dichorionic and monochorionic twin pregnancies, with the exception of EFW in monochorionic twins. The crossover points, where the twin 50^th^ centiles cross from above the singleton 50^th^ centile to below it, occur at approximately 28 weeks' gestation for dichorionic twins. The crossover points for monochorionic twins occur earlier in gestation, with the exception of EFW, which never goes above the singleton 50^th^ centile.

### Comparison with twin reference ranges

**Figure 2:**
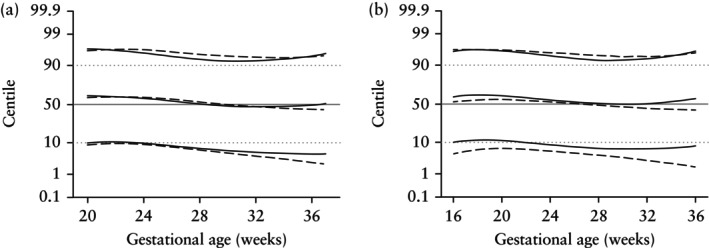
Graphs showing comparison of 10^th^, 50^th^ and 90^th^ centiles for estimated fetal weight, based on reference range determined in this study (using ESPRiT data) (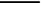) and STORK^24^, ^25^ twin references (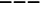), with the FMF twin reference^21^, in dichorionic (a) and monochorionic (b) twins. FMF centiles are shown in gray.

Comparisons of this study's results and the STORK twin reference^25^ with the FMF twin reference^21^ for EFW are presented in Figure 2. Relative to Figure 1d, there is considerable agreement between the three studies for the 50^th^ centiles. The 10^th^ centile for dichorionic twins in this study and the STORK study depart from the FMF reference in the third trimester. For monochorionic twins, the 10^th^ centile determined in this study is more consistent with the FMF twin reference.

### Association of fetal biometry and perinatal outcomes

In this analysis, we used the final predelivery ultrasound examination to determine the association between fetal biometry centile and adverse perinatal outcome. We used outcome prevalence as our centile cut‐offs (the *p* ^*th*^ biometry centile where *p* is prevalence of outcome, as presented in Table 2) in the comparisons of reference ranges. The 13^th^ and 23^rd^ biometry centiles were used in assessment of composite adverse perinatal outcome in dichorionic and monochorionic twins, respectively. The 19^th^ and 31^st^ biometry centiles were used for NICU admissions, the 12^th^ and 24^th^ biometry centiles for preterm delivery and the 9^th^ and 10^th^ biometry centiles for birth‐weight discordance ≥ 25%, in dichorionic and monochorionic twins, respectively.

The association between fetal biometry and perinatal outcome for dichorionic and monochorionic twins is presented in Tables 3 and 4, respectively. The relevant *n*‐values are provided and test positivity relates to the number falling under the *p* ^th^ biometry centile at the last ultrasound examination. For composite adverse perinatal outcome in dichorionic twins, the OR associated with EFW was 2.0 (95% CI, 1.3–3.0) using the reference range developed in this study, 3.1 (95% CI, 2.0–4.8) using the STORK reference, 1.9 (95% CI, 1.3–2.8) using the FMF twin reference and 1.0 (95% CI, 0.7–1.4) using the FMF singleton reference. For monochorionic twins, the equivalent OR was 3.0 (95% CI, 1.6–5.5) for our reference, 5.6 (95% CI, 3.0–10.6) for the STORK reference, 3.5 (95% CI, 2.0–6.2) for the FMF twin reference and 1.4 (95% CI, 0.8–2.4) for the FMF singleton reference.

**Table 3:** Association between fetal biometry and perinatal outcome in dichorionic twins, using outcome‐based prevalence for biometry centile cut‐offs at last ultrasound examination before delivery

ESPRiT study outcome/ reference range	Outcome/test positive	Outcome/test negative	OR (95% CI)	Perinatal death (*n* = 4)
*CAPO (13.3% prevalence)*				
EFW				
ESPRiT twin	42/197	164/1355	2.0 (1.3–3.0)^a^	1
STORK twin*	40/138	166/1414	3.1 (2.0–4.8)^c^	1
FMF twin†	57/283	149/1269	1.9 (1.3–2.8)^b^	1
FMF singleton‡	75/572	131/980	1.0 (0.7–1.4)	1
AC				
ESPRiT twin	43/215	163/1337	1.8 (1.2–2.7)^a^	1
STORK twin*	35/151	171/1401	2.2 (1.4–3.3)^b^	1
FMF singleton‡	40/254	166/1298	1.3 (0.8–1.9)	1
HC				
ESPRiT twin	33/236	173/1316	1.1 (0.7–1.6)	0
STORK twin*	47/354	159/1198	1.0 (0.7–1.5)	1
FMF singleton‡	45/345	161/1207	1.0 (0.7–1.4)	0
FL				
ESPRiT twin	43/216	163/1336	1.8 (1.2–2.7)^a^	2
STORK twin*	49/213	157/1339	2.2 (1.5–3.4)^c^	2
FMF singleton‡	45/286	161/1266	1.3 (0.9–1.9)	0
*NICU admission (≥ 10 days) (18.6% prevalence)*				
EFW				
ESPRiT twin	120/287	206/1265	3.7 (2.7–5.0)^c^	1
STORK twin*	106/239	220/1313	4.0 (2.9–5.4)^c^	1
FMF twin†	130/364	196/1188	2.8 (2.1–3.8)^c^	1
FMF singleton‡	172/672	154/880	1.6 (1.2–2.1)^b^	1
AC				
ESPRiT twin	106/295	220/1257	2.6 (2.0–3.5)^c^	1
STORK twin*	86/208	240/1344	3.2 (2.3–4.5)^c^	1
FMF singleton‡	122/387	204/1165	2.2 (1.6–2.9)^c^	1
HC				
ESPRiT twin	90/344	236/1208	1.5 (1.1–2.0)	1
STORK twin*	118/491	208/1061	1.3 (1.0–1.7)	1
FMF singleton‡	126/498	200/1054	1.4 (1.1–1.9)^a^	0
FL				
ESPRiT twin	84/292	242/1260	1.7 (1.2–2.4)^a^	2
STORK twin*	95/295	231/1257	2.1 (1.5–2.9)^c^	2
FMF singleton‡	107/405	219/1147	1.5 (1.1–2.0)^a^	3
*PTD < 34 weeks' gestation (12.4% prevalence)*				
EFW				
ESPRiT twin	33/156	63/620	2.4 (1.5–3.8)^b^	1
STORK twin*	32/111	64/665	3.8 (2.3–6.2)^c^	1
FMF twin†	39/217	57/559	1.9 (1.2–3.0)^a^	1
FMF singleton‡	48/396	48/380	1.0 (0.6–1.5)	1
AC				
ESPRiT twin	35/175	61/601	2.2 (1.4–3.5)^b^	1
STORK twin*	30/123	66/653	2.9 (1.8–4.7)^c^	1
FMF singleton‡	31/196	65/580	1.5 (0.9–2.4)	1
HC				
ESPRiT twin	23/190	73/586	1.0 (0.6–1.6)	0
STORK twin*	35/269	61/507	1.1 (0.7–1.7)	1
FMF singleton‡	32/251	64/525	1.1 (0.7–1.7)	0
FL				
ESPRiT twin	32/167	64/609	2.0 (1.3–3.2)^a^	2
STORK twin*	37/159	59/617	2.9 (1.8–4.5)^c^	2
FMF singleton‡	33/211	63/565	1.5 (0.9–2.3)	0
*BW discordance ≥ 25% (8.5% prevalence)*				
EFW				
ESPRiT twin	47/245	19/531	6.4 (3.7–11.2)^c^	1
STORK twin*	45/212	21/564	7.0 (4.0–12.0)^c^	1
FMF twin†	48/290	18/486	5.2 (2.9–9.1)^c^	1
FMF singleton‡	57/479	9/297	4.3 (2.1–8.9)^c^	1
AC				
ESPRiT twin	46/247	20/529	5.8 (3.4–10.1)^c^	1
STORK twin*	43/191	23/585	7.1 (4.1–12.2)^c^	1
FMF singleton‡	48/326	18/450	4.1 (2.4–7.3)^c^	1
HC				
ESPRiT twin	44/301	22/475	3.5 (2.1–6.0)^c^	1
STORK twin*	50/404	16/372	3.1 (1.8–5.6)^b^	1
FMF singleton‡	49/402	17/374	2.9 (1.6–5.2)^b^	0
FL				
ESPRiT twin	39/252	27/524	3.4 (2.0–5.6)^c^	2
STORK twin*	40/254	26/522	3.6 (2.1–6.0)^c^	2
FMF singleton‡	44/332	22/444	2.9 (1.7–5.0)^c^	2

Data are given as *n*/*N* or *n*, unless stated otherwise. Individual twins were analyzed for composite adverse perinatal outcome (CAPO) and neonatal intensive care unit (NICU) admission. The smaller of a twin pair, for the biometry parameter considered, was used in analysis of preterm delivery (PTD) and birth‐weight (BW) discordance ≥ 25%. *P*‐values were calculated using logistic regression/generalized estimating equation analysis.

* Stirrup *et al*.^24^ (abdominal circumference (AC), head circumference (HC) and femur diaphysis length (FL)); Stirrup *et al*.^25^ (estimated fetal weight (EFW)).

† Wright *et al*.^21^.

‡ Snijders and Nicolaides^9^ (AC, HC and FL); Nicolaides *et al*.^12^ (EFW).

^a^ *P* < 0.01.

^b^ *P* < 0.001.

^c^ *P* < 0.0001. ESPRiT, Evaluation of Sonographic Predictors of Restricted growth in Twins; FMF, Fetal Medicine Foundation; OR, odds ratio; STORK, Southwest Thames Obstetric Research Collaborative.

**Table 4:** Association between fetal biometry and perinatal outcome in monochorionic twins, using outcome‐based prevalence for biometry centile cut‐offs at last ultrasound examination before delivery

ESPRiT study outcome/ reference range	Outcome/test positive	Outcome/test negative	OR (95% CI)	Perinatal death (*n* = 7)
*CAPO (23.3% prevalence)*				
EFW				
ESPRiT twin	31/77	49/267	3.0 (1.6–5.5)^b^	4
STORK twin*	28/51	52/293	5.6 (3.0–10.6)^c^	4
FMF twin†	36/86	44/258	3.5 (2.0–6.2)^c^	4
FMF singleton‡	50/194	30/150	1.4 (0.8–2.4)	5
AC				
ESPRiT twin	32/77	48/267	3.2 (1.9–5.7)^c^	3
STORK twin*	27/48	53/296	5.9 (3.0–11.6)^c^	3
FMF singleton‡	38/125	42/219	1.8 (1.1–3.1)	5
HC				
ESPRiT twin	21/72	59/272	1.5 (0.8–2.7)	3
STORK twin*	26/112	54/232	1.0 (0.6–1.8)	3
FMF singleton‡	37/142	43/202	1.3 (0.8–2.3)	5
FL				
ESPRiT twin	30/86	50/258	2.2 (1.2–4.0)^a^	4
STORK twin*	29/67	51/277	3.4 (1.8–6.3)^b^	4
FMF singleton‡	41/147	39/197	1.6 (0.9–2.7)	4
*NICU admission (≥ 10 days) (30.8% prevalence)*				
EFW				
ESPRiT twin	54/101	62/243	3.4 (2.0–5.7)^c^	4
STORK twin*	47/74	69/270	5.1 (2.7–9.5)^c^	4
FMF twin†	56/101	60/243	3.8 (2.2–6.5)^c^	4
FMF singleton‡	83/218	33/126	1.7 (1.0–3.0)	5
AC				
ESPRiT twin	55/110	61/234	2.8 (1.7–4.6)^c^	5
STORK twin*	44/77	72/267	3.6 (2.0–6.4)^c^	4
FMF singleton‡	71/171	45/173	2.0 (1.2–3.4)^a^	5
HC				
ESPRiT twin	39/98	77/246	1.5 (0.9–2.4)	3
STORK twin*	49/145	67/199	1.0 (0.6–1.7)	4
FMF singleton‡	70/185	46/159	1.5 (0.9–2.5)	5
FL				
ESPRiT twin	42/110	74/234	1.3 (0.8–2.2)	4
STORK twin*	44/102	72/242	1.8 (1.1–3.0)	4
FMF singleton‡	70/186	46/158	1.5 (0.9–2.4)	4
*PTD < 34 weeks' gestation (24.4% prevalence)*				
EFW				
ESPRiT twin	27/67	15/105	4.0 (1.9–8.4)^b^	3
STORK twin*	24/47	18/125	6.2 (2.9–13.3)^c^	3
FMF twin†	29/71	13/101	4.7 (2.2–9.9)^c^	3
FMF singleton‡	32/127	10/45	1.2 (0.5–2.6)	3
AC				
ESPRiT twin	24/67	18/105	2.7 (1.3–5.5)^a^	3
STORK twin*	21/46	21/126	4.2 (2.0–8.9)^b^	3
FMF singleton‡	27/96	15/76	1.6 (0.8–3.3)	3
HC				
ESPRiT twin	19/63	23/109	1.6 (0.8–3.3)	2
STORK twin*	21/86	21/86	1.0 (0.5–2.0)	2
FMF singleton‡	25/102	17/70	1.0 (0.5–2.1)	4
FL				
ESPRiT twin	25/72	17/100	2.6 (1.3–5.3)^a^	3
STORK twin*	23/57	19/115	3.4 (1.7–7.0)^b^	3
FMF singleton‡	30/105	12/67	1.8 (0.9–3.9)	3
*BW discordance ≥ 25% (9.9% prevalence)*				
EFW				
ESPRiT twin	15/62	2/110	17 (3.8–78.4)^b^	3
STORK twin*	13/45	4/127	13 (3.8–40.9)^c^	3
FMF twin†	15/70	2/102	14 (3.0–61.8)^b^	3
FMF singleton‡	16/125	1/47	6.8 (0.9–52.4)	3
AC				
ESPRiT twin	14/63	3/109	10 (2.8–36.7)^b^	3
STORK twin*	12/41	5/131	10 (3.4–31.9)^c^	3
FMF singleton‡	14/94	3/78	4.4 (1.2–15.8)	3
HC				
ESPRiT twin	8/57	9/115	1.9 (0.7–5.3)	2
STORK twin*	10/84	7/88	1.6 (0.6–4.3)	2
FMF singleton‡	13/102	4/70	2.4 (0.8–7.7)	4
FL				
ESPRiT twin	12/71	5/101	3.9 (1.3–11.6)	3
STORK twin*	12/55	5/117	6.3 (2.1–18.8)^a^	3
FMF singleton‡	15/104	2/68	5.6 (1.2–25.2)	3

Data are given as *n*/*N* or *n*, unless stated otherwise. Individual twins were analyzed for composite adverse perinatal outcome (CAPO) and neonatal intensive care unit (NICU) admission. The smaller of a twin pair, for the biometry parameter considered, was used in analysis of preterm delivery (PTD) and birth‐weight (BW) discordance ≥ 25%. *P*‐values were calculated using logistic regression/generalized estimating equation analysis.

* Stirrup *et al*.^24^ (abdominal circumference (AC), head circumference (HC) and femur diaphysis length (FL)); Stirrup *et al*.^25^ (estimated fetal weight (EFW)).

† Wright *et al*.^21^.

‡ Snijders and Nicolaides^9^ (AC, HC and FL); Nicolaides *et al*.^12^ (EFW).

^a^ *P* < 0.01.

^b^ *P* < 0.001.

^c^ *P* < 0.0001. ESPRiT, Evaluation of Sonographic Predictors of Restricted growth in Twins; FMF, Fetal Medicine Foundation; OR, odds ratio; STORK, Southwest Thames Obstetric Research Collaborative.

## DISCUSSION

### Summary of main findings

A total of 15 274 fetal ultrasound assessments across 948 twin pregnancies were obtained in the ESPRiT study, with complete ultrasound biometry measures (EFW, AC, HC, FL and BPD) and adverse perinatal outcomes, ascertained in this prospective cohort study of unselected twin pregnancies. Our analysis was novel with regard to the comparisons of AC, HC and FL, which also showed a strong wave‐like pattern that differed from that in singletons.

We demonstrated that a twin reference range can be used successfully in the prediction of composite adverse perinatal outcome, NICU admission (≥ 10 days), preterm delivery and intertwin birth‐weight discordance ≥ 25% in both monochorionic and dichorionic twin pregnancies. We evaluated fetal biometry centiles using thresholds that were reflective of the prevalence of commonly considered adverse perinatal outcomes. The perinatal mortality rates were low in this study population but there was no evidence that the prediction of perinatal mortality is compromised when prevalence is considered in the setting of centile thresholds for other adverse outcomes.

In the evaluation of outcomes occurring in this study, we found similarities in the risk of adverse perinatal outcomes using our study centile estimates with those determined from other large‐scale twin studies, specifically the STORK study^24^, ^25^ and the FMF standard for twins^21^. In contrast, the FMF singleton references^9^, ^12^ did not show similar elevated risks that were found using twin references.

Finally, it is noteworthy that, for all twins combined, the 10^th^ centile for birth weights in this study corresponded to the 15^th^ centile on a twin reference chart^28^ and the 0.5^th^ centile on the FMF singleton reference^12^. This extreme disparity was evident for both monochorionic and dichorionic twins.

### Clinical and research implications

Published reference ranges for twins vary with respect to the biometric measurements included and the analytical approach. Some studies^14^, ^15^, ^16^, ^17^ report a combined dichorionic and monochorionic twin reference. Consequently, it cannot be determined from these studies whether references should be separated according to chorionicity. Other studies^18^, ^19^, ^20^, ^21^ present a reference range for EFW but do not present complimentary references for its constituent parts (AC, HC, FL with/without BPD). Many studies^14^, ^15^, ^16^, ^18^, ^19^, ^20^, ^22^, ^23^ do not provide a complete method for exact centile calculation. The STORK study^24^, ^25^, and the ESPRiT study, are exceptions and additionally evaluated adverse perinatal outcomes in their study populations. It is acknowledged that other twin studies, though smaller in scale, have merits of their own. The Eunice Kennedy Shriver National Institute of Child Health and Human Development (NICHD) Fetal Growth Studies^23^ compared differences in fetal growth between dichorionic twins and singletons, from the same underlying baseline population, weighted for racial/ethnicity distribution. It is noteworthy that the departure from singleton centiles for EFW observed in our dichorionic twin population (Figure 1d) is remarkably similar to the pattern observed in the NICHD twin study^23^ (illustrated in figure 5 of the FMF twin study).

ISUOG guidance^30^ states that twin‐specific growth charts have the potential to reduce unnecessary medical intervention, but concerns remain in relation to under‐recognition of reduced growth in the third trimester attributable to placental insufficiency. Nonetheless, the STORK study indicated that, when compared with use of a singleton reference, a twin reference could safely reduce unnecessary medical intervention in twin pregnancy^35^. More recently, Prasad and Khalil^36^ have called for the implementation of twin‐specific charts, aimed at avoiding overdiagnosis of intrauterine growth restriction in twin pregnancies. Hiersch *et al*.^37^, in a review of the currently available evidence, suggested that the use of twin charts is reasonable and may be preferred over the use of singleton charts when monitoring the growth of twin fetuses, concluding that further evidence is required and that clinical trials are a possible way forward. The study by Dall'Asta *et al*.^38^ shares the same opinion and specifically raises the concern that thresholds for growth in twins should not be considered the same as for singletons.

### Strengths and limitations

The primary strength of the ESPRiT study was the prospective recruitment and assessment of twin pregnancies, thus minimizing potential bias associated with patient selection or reporting of outcome data. In addition, the dedicated study personnel, high adherence of participants to the study procedures, large sample size and completeness of the pregnancy outcome data were notable strengths.

This study has several limitations. The relative homogeneity of the population ethnicity (predominantly Caucasian), the high rate of NICU admissions and the arbitrary cut‐off for the number of days admitted, and the prevalence rates of adverse outcomes may not be indicative of other populations. Consequently, though internal validity is very strong, the external validation and applicability of our results may not hold in other, more diverse populations.

### Conclusions

The use of an inappropriate reference standard might erroneously indicate that an abnormally grown twin pregnancy lies within a reference range. This concern holds true for singleton pregnancy as it does for twin. However, given the large sample sizes and strong methodology underpinning several proposed twin reference ranges, and a general consistency in relation to the different growth patterns observed in twin pregnancy, this study adds to the available evidence that a twin‐specific reference range may find application in clinical practice.

Knowledge gaps prevail around choice of birth‐weight centile chart, definition of intrauterine growth restriction, and choice of cut‐offs for fetal biometry. The gap in knowledge around relevant centile cut‐offs is important and consideration of prevalence might guide us in the use of a twin‐based reference in practice.

This study strengthens the case for the implementation of specific reference ranges for fetal growth evaluation in twin pregnancy, and that outcome prevalence is a vital component in further ascertainment of the value of twin reference charts in clinical practice.

## Supporting information


**Appendix S1** Statistical modeling of fetal biometry for construction of twin growth reference charts

**Figure S1** Scatter‐density plots showing dichorionic twin fetal biometry data in ESPRiT study.

**Figure S2** Scatter‐density plots showing monochorionic twin fetal biometry data in ESPRiT study.

**Table S1** Fetal biometry centiles for dichorionic twin pregnancy, established using ESPRiT study data

**Table S2** Fetal biometry centiles for monochorionic twin pregnancy, established using ESPRiT study data

## Data Availability

Research data are not shared.
