# Supplementary material for: Fetal biometry reference ranges derived from prospective twin population and evaluation of adverse perinatal outcome
Source: Ultrasound Obstet Gynecol. 2025 Feb 27;65(4):436–46. doi: 10.1002/uog.29190 (PMC11961106; doi:10.1002/uog.29190)
Supplement: Supplementary file 1 — Appendix S1 Statistical modeling of fetal biometry for construction of twin growth reference charts [file UOG-65-436-s001.docx]

Statistical Modelling of Fetal Biometry

We chose a parametric approach for fetal biomety modelling rather than a non-parametric approach as the former provides a mechanism for individual centile determination. We modelled mean profiles and variance structures, separately, to determine equations for z-score and centile determination. The parametric models are longitudinal and this modelling approach implies that preterm deliveries provide information on reference values relevant to later gestations.

For the mean profiles, we used fractional polynomials with a default set of powers: −2, −1, −0.5, 0, 0.5, 1, 2, 3, with 0 signifying natural log. Two-degree fractional polynomials (FP) are usually sufficient for any setting. The log-transform was used first and Box-Cox transforms did not indicate a better alternative. Consequently, the mean value of a singular twin biometric measure at time *t* may be expressed generically as:

$E\left( log(Y | t) \right){=\beta_{0}+\beta}_{1}\times t^{p1}+\beta_{2}\times t^{p2}$ (1)

Using the FP mean model (1), we then modeled variance using random coefficient regression, in a linear mixed model with fixed mean term without intercept. This approach is very challenging due to significant measurement error in fetal biometry. Few terms are usually chosen (e.g. a quadratic, or a cubic excluding the quadratic term), potentially due to model converge issues (see below). In the course of modelling our data, we could not fit more than two terms plus intercept. Given the variation in fetal growth trajectories, such three-term models may have low predictive ability, irrespective of choice of polynomial. Additionally, random coefficients are usually highly correlated, necessitating unstructured variance structures. Consequently, 3 term random coefficient models will have 6 variance components and (if error variance and variation in the hierarchy is included, e.g. pregnancy) then this results in at least 8 variance components to describe the overall variance structure.

We sought an alternative approach to allow greater flexibility in modelling individual fetal trajectories. Individualised Growth Assessment (IGA) was used successfully in a sub-cohort of our study population (those having an ultrasound examination within 3 days of delivery)^33^. Rossavik growth models are used, and most commonly associated with, IGA. In the IGA context, 2^nd^ trimester ultrasound measurements are used to obtain a growth pathology score in the third trimester.

Rossvaik models represent a specific class of parametric-spline-growth models. Their implementation here, in the context of hierarchical models and developing reference ranges, is novel and quite different to their use in IGA. Rossavik models are of the form:

$log(Y|t)=a+b\times log\left( t \right)+c\times t\times log(t)$ (2)

The Rossavik coefficients in our study for all biometric measures and for both monochorionic and dichorionic were normally distributed – critically important for the mean+SD approach to centile estimation. In our modelling approach we used Rossavik models at the fetus level, not the pregnancy level, and used a simple random intercept for the latter. This approach accords with fetal growth discordance and, for pregnancies uncomplicated by discordant growth, it seems reasonable that fetuses might follow a parallel path. The resulting model has 8 variance components (6 from the unstructured covariance matrix for the Rossavik components and a pregnancy-level and residual error component). More complex pregnancy-level models, without simplifying the fetus-level models, failed to converge in our modelling approach. This challenge persisted even when simpler polynomial models, rather than Rossavik models, were used.

A key factor in using fractional polynomials and random Rossavik coefficients for our fetal biometry data was the fact that we had fetal ultrasound data from 12 weeks (aiding the fit of fractional polynomials to determine centiles from 16 weeks) and the median number of ultrasound assessments was 8 (facilitating well-behaved Rossavik models, which can give unrealistic growth projections when used with fewer data points).

Prior to fitting Rossavik models, we assessed the FP models for gross outliers, |z|> 4.5 (1 in 62,564), removed and re-fitted in a single iteration. This resulted in <10 data points removed for each biometric measure.

The resulting mean models and variance formulas for the log-transformed data are presented in Table B1. These may be to used to determine z-score and centiles.

The software packages used were: SAS v9.4 (data management, statistical summaries, linear mixed models, table generation), STATA SE 18.5 (fractional polynomials) and R 4.4.1 (graphics, ggplot).

**Table B1. Mean & variance models for log-transformed data, expressed in generic code**

| Biometry | | t=GA/10, where GA is gestational age in week units  **=power, *=multiplication, log is natural log |
| --- | --- | --- |
| Dichorionic Twins | | |
| AC | Mean | 7.617518 -0.6301568*(t**-2) -3.46944*(t**-0.5) |
|  | Variance | 0.014716 -0.046291*log(t) +0.046728*(log(t)**2) +0.007143*t*log(t) +0.001553960*(t*log(t))**2 -0.016120*t*(log(t)**2) |
| HC | Mean | 6.579418 -2.832467*(t**-1) -0.0006039*(t**3) |
|  | Variance | 0.009898 -0.030902*log(t) +0.031322*(log(t)**2) +0.004603*t*log(t) +0.001002830*(t*log(t))**2 -0.010640*t*(log(t)**2) |
| FL | Mean | 3.958543 -3.09981*(t**-2) +0.1375774*t |
|  | Variance | 0.028162 -0.091563*log(t) +0.084595*(log(t)**2) +0.014197*t*log(t) +0.002348121*(t*log(t))**2 -0.027585*t*(log(t)**2) |
| BPD | Mean | 10.25267 -7.747729*(t**-0.5)-1.319771*log(t) |
|  | Variance | 0.013933 -0.047814*log(t) +0.052712*(log(t)**2) +0.007698*t*log(t) +0.001882119*(t*log(t))**2 -0.019060*t*(log(t)**2) |
| EFW | Mean | -2.644686 +6.087399*(t**0.5) -0.0226155*(t**3) |
|  | Variance | 0.019065 -0.045605*log(t) +0.073594*(log(t)**2) +0.006191*t*log(t) +0.004016706*(t*log(t))**2 -0.030412*t*(log(t)**2) |
| Monochorionic Twins | | |
| AC | Mean | 6.214562 -2.759548*(t**-1) + 0.0801721*t |
|  | Variance | 0.014410 -0.049936*log(t) +0.05990*(log(t)**2) +0.008195*t*log(t) +0.002286592*(t*log(t))**2 -0.022397*t*(log(t)**2) |
| HC | Mean | 9.021217 -4.997093*(t**-0.5) -0.1740898*t |
|  | Variance | 0.007973 -0.026049*log(t) +0.02941*(log(t)**2) +0.004293*t*log(t) +0.001085237*(t*log(t))**2 -0.010833*t*(log(t)**2) |
| FL | Mean | 3.72931 -2.639292*(t**-2) +0.5292293*log(t) |
|  | Variance | 0.028166 -0.095078*log(t) +0.09451*(log(t)**2) +0.015261*t*log(t) +0.002814907*(t*log(t))**2 -0.032026*t*(log(t)**2) |
| BPD | Mean | 4.325112 -1.815061*(t**-0.5) + 1.646903*(t**-0.5)*log(t) |
|  | Variance | 0.009365 -0.030940*log(t) +0.03619*(log(t)**2) +0.004845*t*log(t) +0.001289008*(t*log(t))**2 -0.012981*t*(log(t)**2) |
| EFW | Mean | -2.589142 + 6.016928*(t**0.5) -0.0213112*(t**3) |
|  | Variance | 0.021279 -0.059759*log(t) +0.10280*(log(t)**2) +0.007805*t*log(t) +0.004783167*(t*log(t))**2 -0.039636*t*(log(t)**2) |

**Example: Calculation of centiles for AC at 36 weeks in dichorionic twins.**

From table B1, the mean at 36 weeks (t=3.6) is expressed as:

7.617518 -0.6301568*(3.6**-2) -3.46944*(3.6**-0.5)

= 5.740339

The variance at 36 weeks is expressed as:

0.014716 -0.046291*log(3.6) +0.046728*(log(3.6)**2) +0.007143*3.6*log(3.6) +0.001553960*(3.6*log(3.6))**2 -0.016120*3.6*(log(3.6)**2)

= 0.00285617

The standard deviation (square root) is thus 0.05344315

(1) The 10^th^ centile can be calculated using Z_10th_ score = -1.28155, and expressed as:

exp(5.740339 -1.28155*0.05344315)

= 290.5713, or 291mm, as given in Table A1 of appendix A.

(2) Z-scores and percentile may be obtained for individual fetus measurements. For example, using the mean and standard deviation calculated above, the z-score for a dichorionic fetus with an AC of 320mm at 36 weeks is:

Z= (log(320) - 5.740339) / 0.05344315 = 0.52 and corresponds to the 70^th^ percentile.
