# Supplementary figures and images for: Fetal biometry reference ranges derived from prospective twin population and evaluation of adverse perinatal outcome
Source: Ultrasound Obstet Gynecol. 2025 Feb 27;65(4):436–46. doi: 10.1002/uog.29190 (PMC11961106; doi:10.1002/uog.29190)

**Figure S1** Scatter-density plots showing dichorionic twin fetal biometry data in ESPRiT study

**
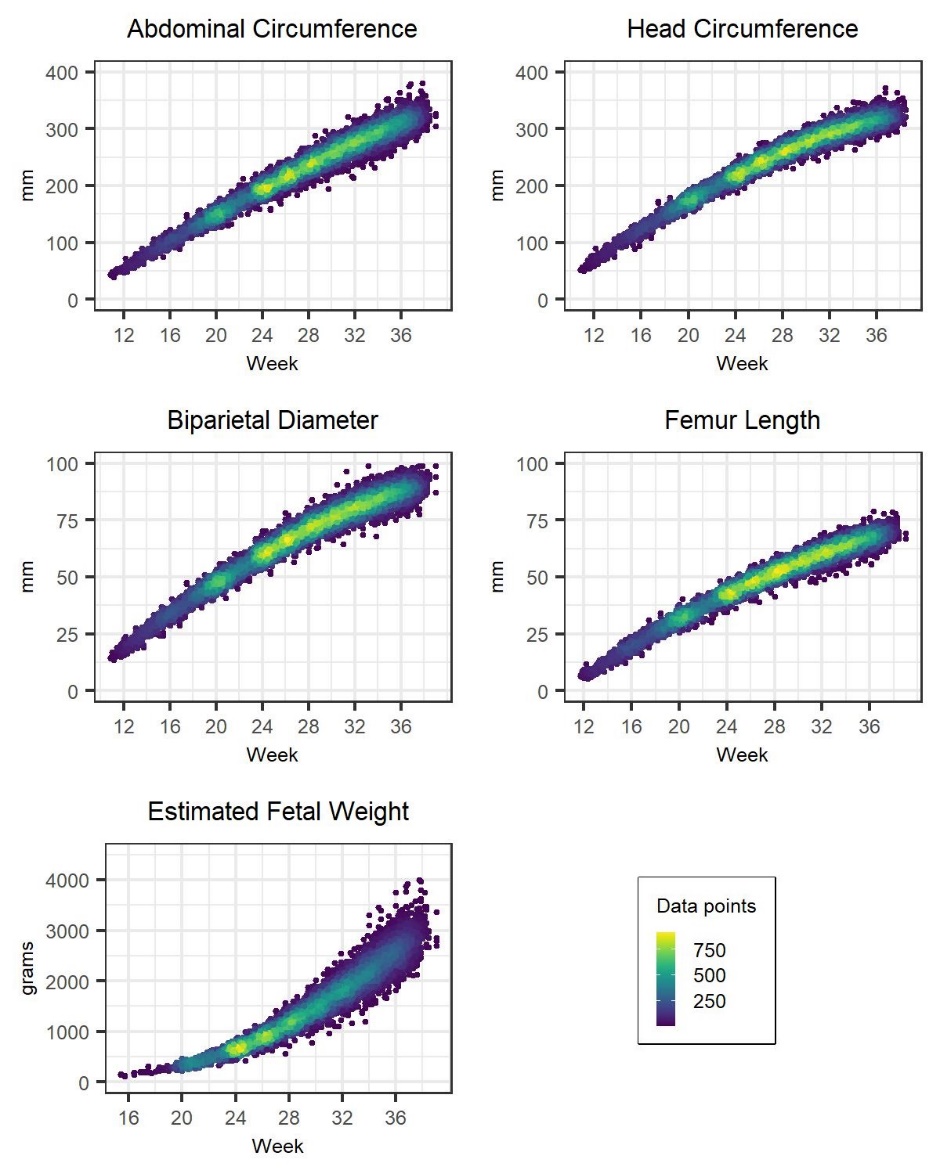
**

Supplement: Supplementary file 2 — Figure S1 Scatter‐density plots showing dichorionic twin fetal biometry data in ESPRiT study. [file UOG-65-436-s002.docx]
