# Supplementary material for: Fetal biometry reference ranges derived from prospective twin population and evaluation of adverse perinatal outcome
Source: Ultrasound Obstet Gynecol. 2025 Feb 27;65(4):436–46. doi: 10.1002/uog.29190 (PMC11961106; doi:10.1002/uog.29190)
Supplement: Supplementary file 3 — Figure S2 Scatter‐density plots showing monochorionic twin fetal biometry data in ESPRiT study. [file UOG-65-436-s004.docx]

**Figure S2** Scatter-density plots showing monochorionic twin fetal biometry data in ESPRiT study

**
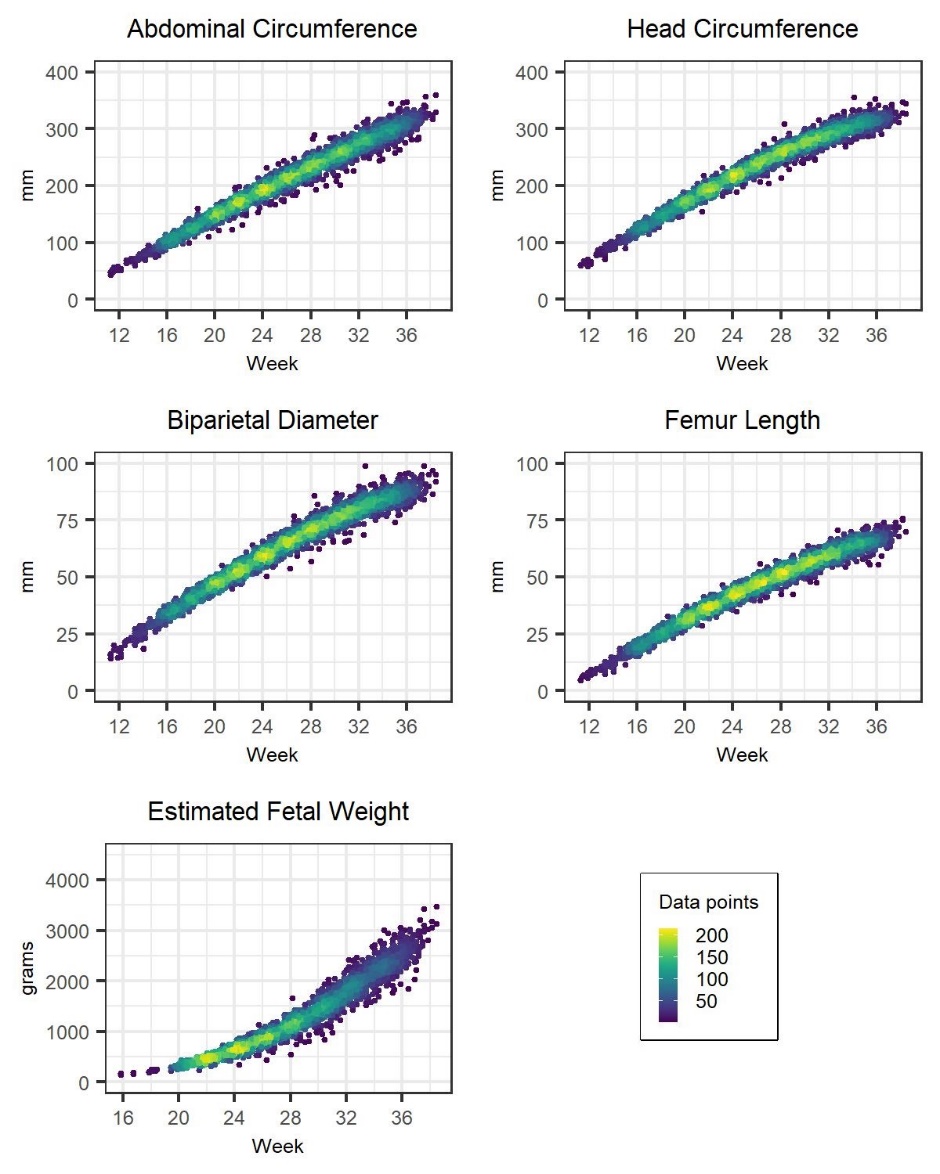
**
