# Supplementary material for: Fetal biometry reference ranges derived from prospective twin population and evaluation of adverse perinatal outcome
Source: Ultrasound Obstet Gynecol. 2025 Feb 27;65(4):436–46. doi: 10.1002/uog.29190 (PMC11961106; doi:10.1002/uog.29190)
Supplement: Supplementary file 4 — Table S1 Fetal biometry centiles for dichorionic twin pregnancy, established using ESPRiT study data [file UOG-65-436-s003.docx]

**Table S1** Fetal biometry centiles for dichorionic twin pregnancy, established using ESPRiT study data

| *GA (week)* | *Abdominal*  *Circumference*  *(mm)* | | | *Head*  *Circumference*  *(mm)* | | | *Femur*  *Length*  *(mm)* | | | *Biparietal*  *Diameter*  *(mm)* | | | *Estimated*  *Fetal Weight*  *(grams)* | | |
| --- | --- | --- | --- | --- | --- | --- | --- | --- | --- | --- | --- | --- | --- | --- | --- |
|  | *10th* | *50th* | *90th* | *10th* | *50th* | *90th* | *10th* | *50th* | *90th* | *10th* | *50th* | *90th* | *10th* | *50th* | *90th* |
| 16 | 95 | 102 | 111 | 115 | 122 | 131 | 17.6 | 19.4 | 21.5 | 31.0 | 33.4 | 35.9 | 126 | 143 | 163 |
| 17 | 106 | 114 | 123 | 128 | 136 | 144 | 20.7 | 22.6 | 24.8 | 34.6 | 37.0 | 39.5 | 157 | 178 | 202 |
| 18 | 118 | 126 | 135 | 141 | 149 | 157 | 23.8 | 25.8 | 28.0 | 38.0 | 40.5 | 43.2 | 193 | 219 | 249 |
| 19 | 129 | 138 | 147 | 153 | 161 | 170 | 26.7 | 28.8 | 31.1 | 41.4 | 44.0 | 46.8 | 236 | 268 | 304 |
| 20 | 140 | 149 | 159 | 165 | 174 | 183 | 29.6 | 31.8 | 34.1 | 44.7 | 47.4 | 50.3 | 286 | 325 | 369 |
| 21 | 151 | 161 | 171 | 177 | 186 | 195 | 32.4 | 34.6 | 37.0 | 47.9 | 50.8 | 53.8 | 344 | 390 | 444 |
| 22 | 162 | 172 | 183 | 188 | 197 | 207 | 35.0 | 37.4 | 39.9 | 51.0 | 54.0 | 57.1 | 409 | 466 | 530 |
| 23 | 172 | 183 | 195 | 199 | 209 | 219 | 37.6 | 40.0 | 42.6 | 54.0 | 57.1 | 60.4 | 484 | 551 | 627 |
| 24 | 183 | 194 | 206 | 209 | 219 | 230 | 40.0 | 42.5 | 45.2 | 56.8 | 60.1 | 63.6 | 568 | 648 | 738 |
| 25 | 193 | 205 | 218 | 219 | 230 | 241 | 42.4 | 45.0 | 47.8 | 59.6 | 63.0 | 66.6 | 662 | 755 | 861 |
| 26 | 203 | 215 | 229 | 229 | 240 | 251 | 44.6 | 47.4 | 50.3 | 62.3 | 65.8 | 69.6 | 766 | 874 | 998 |
| 27 | 213 | 226 | 240 | 238 | 249 | 261 | 46.8 | 49.6 | 52.7 | 64.8 | 68.5 | 72.4 | 879 | 1005 | 1149 |
| 28 | 222 | 236 | 251 | 247 | 258 | 271 | 48.9 | 51.9 | 55.0 | 67.3 | 71.1 | 75.1 | 1002 | 1147 | 1313 |
| 29 | 231 | 246 | 261 | 255 | 267 | 280 | 50.9 | 54.0 | 57.3 | 69.6 | 73.5 | 77.7 | 1134 | 1300 | 1490 |
| 30 | 241 | 256 | 272 | 263 | 276 | 289 | 52.9 | 56.1 | 59.5 | 71.9 | 75.9 | 80.2 | 1274 | 1463 | 1681 |
| 31 | 249 | 265 | 282 | 271 | 284 | 297 | 54.8 | 58.1 | 61.6 | 74.0 | 78.2 | 82.6 | 1420 | 1635 | 1883 |
| 32 | 258 | 275 | 293 | 278 | 291 | 305 | 56.7 | 60.1 | 63.7 | 76.1 | 80.4 | 84.9 | 1572 | 1815 | 2096 |
| 33 | 267 | 284 | 303 | 285 | 299 | 313 | 58.5 | 62.0 | 65.8 | 78.0 | 82.4 | 87.1 | 1726 | 2000 | 2317 |
| 34 | 275 | 293 | 313 | 292 | 306 | 320 | 60.3 | 64.0 | 67.8 | 79.8 | 84.4 | 89.3 | 1882 | 2189 | 2546 |
| 35 | 283 | 302 | 323 | 298 | 312 | 328 | 62.1 | 65.8 | 69.8 | 81.5 | 86.3 | 91.4 | 2036 | 2378 | 2779 |
| 36 | 291 | 311 | 333 | 304 | 319 | 334 | 63.8 | 67.7 | 71.8 | 83.1 | 88.1 | 93.5 | 2185 | 2566 | 3014 |
| 37 | 298 | 320 | 343 | 309 | 325 | 341 | 65.4 | 69.5 | 73.8 | 84.5 | 89.8 | 95.5 | 2328 | 2749 | 3247 |
| 38 | 305 | 328 | 353 | 314 | 331 | 348 | 67.0 | 71.3 | 75.8 | 85.9 | 91.5 | 97.5 | 2461 | 2925 | 3476 |

GA, exact gestational age in weeks
