# Supplementary material for: Fetal biometry reference ranges derived from prospective twin population and evaluation of adverse perinatal outcome
Source: Ultrasound Obstet Gynecol. 2025 Feb 27;65(4):436–46. doi: 10.1002/uog.29190 (PMC11961106; doi:10.1002/uog.29190)
Supplement: Supplementary file 5 — Table S2 Fetal biometry centiles for monochorionic twin pregnancy, established using ESPRiT study data [file UOG-65-436-s005.docx]

**Table S2** Fetal biometry centiles for monochorionic twin pregnancy, established using ESPRiT study data

| *GA (week)* | *Abdominal*  *Circumference*  *(mm)* | | | *Head*  *Circumference*  *(mm)* | | | *Femur*  *Length*  *(mm)* | | | *Biparietal*  *Diameter*  *(mm)* | | | *Estimated*  *Fetal Weight*  *(grams)* | | |
| --- | --- | --- | --- | --- | --- | --- | --- | --- | --- | --- | --- | --- | --- | --- | --- |
|  | *10th* | *50th* | *90th* | *10th* | *50th* | *90th* | *10th* | *50th* | *90th* | *10th* | *50th* | *90th* | *10th* | *50th* | *90th* |
| 16 | 94 | 101 | 110 | 113 | 121 | 128 | 17.2 | 19.1 | 21.1 | 31.1 | 33.2 | 35.4 | 122 | 139 | 158 |
| 17 | 105 | 113 | 122 | 126 | 133 | 141 | 20.2 | 22.1 | 24.2 | 34.5 | 36.7 | 39.1 | 151 | 173 | 197 |
| 18 | 116 | 125 | 134 | 138 | 146 | 154 | 23.1 | 25.2 | 27.4 | 37.9 | 40.2 | 42.7 | 186 | 213 | 243 |
| 19 | 127 | 136 | 146 | 150 | 158 | 167 | 26.0 | 28.2 | 30.5 | 41.1 | 43.6 | 46.2 | 227 | 259 | 297 |
| 20 | 138 | 148 | 158 | 162 | 171 | 180 | 28.8 | 31.1 | 33.5 | 44.3 | 46.9 | 49.7 | 274 | 314 | 360 |
| 21 | 149 | 159 | 170 | 173 | 183 | 192 | 31.5 | 33.9 | 36.5 | 47.4 | 50.2 | 53.2 | 329 | 377 | 433 |
| 22 | 159 | 170 | 182 | 185 | 194 | 205 | 34.1 | 36.6 | 39.4 | 50.4 | 53.3 | 56.5 | 391 | 450 | 517 |
| 23 | 169 | 181 | 194 | 195 | 206 | 216 | 36.6 | 39.3 | 42.2 | 53.3 | 56.4 | 59.7 | 462 | 532 | 613 |
| 24 | 180 | 192 | 205 | 206 | 217 | 228 | 39.1 | 41.9 | 44.9 | 56.1 | 59.4 | 62.9 | 542 | 625 | 721 |
| 25 | 190 | 203 | 217 | 216 | 227 | 239 | 41.4 | 44.3 | 47.5 | 58.8 | 62.3 | 66.0 | 631 | 729 | 842 |
| 26 | 199 | 213 | 228 | 226 | 237 | 250 | 43.6 | 46.7 | 50.1 | 61.4 | 65.1 | 68.9 | 730 | 844 | 976 |
| 27 | 209 | 223 | 239 | 235 | 247 | 260 | 45.8 | 49.1 | 52.5 | 64.0 | 67.8 | 71.8 | 838 | 971 | 1124 |
| 28 | 219 | 234 | 249 | 244 | 257 | 270 | 47.9 | 51.3 | 54.9 | 66.4 | 70.4 | 74.5 | 957 | 1109 | 1286 |
| 29 | 228 | 244 | 260 | 252 | 266 | 279 | 50.0 | 53.5 | 57.2 | 68.8 | 72.9 | 77.2 | 1084 | 1258 | 1461 |
| 30 | 237 | 253 | 271 | 261 | 274 | 288 | 52.0 | 55.6 | 59.4 | 71.1 | 75.3 | 79.8 | 1219 | 1418 | 1649 |
| 31 | 247 | 263 | 281 | 268 | 282 | 297 | 53.9 | 57.6 | 61.5 | 73.3 | 77.7 | 82.3 | 1363 | 1587 | 1849 |
| 32 | 256 | 273 | 291 | 276 | 290 | 305 | 55.8 | 59.6 | 63.6 | 75.5 | 79.9 | 84.7 | 1512 | 1765 | 2060 |
| 33 | 264 | 282 | 301 | 283 | 298 | 313 | 57.6 | 61.5 | 65.6 | 77.5 | 82.1 | 87.0 | 1666 | 1949 | 2281 |
| 34 | 273 | 292 | 312 | 289 | 305 | 321 | 59.4 | 63.3 | 67.5 | 79.5 | 84.2 | 89.3 | 1823 | 2139 | 2509 |
| 35 | 281 | 301 | 322 | 295 | 311 | 328 | 61.1 | 65.2 | 69.5 | 81.3 | 86.3 | 91.5 | 1980 | 2330 | 2743 |
| 36 | 290 | 310 | 332 | 301 | 318 | 335 | 62.8 | 66.9 | 71.4 | 83.1 | 88.3 | 93.7 | 2134 | 2522 | 2981 |
| 37 | 297 | 319 | 342 | 306 | 324 | 342 | 64.3 | 68.6 | 73.2 | 84.8 | 90.2 | 95.9 | 2284 | 2711 | 3218 |
| 38 | 305 | 328 | 353 | 311 | 329 | 348 | 65.8 | 70.3 | 75.1 | 86.4 | 92.0 | 98.0 | 2426 | 2895 | 3453 |

GA , exact gestational age in weeks
